# Optimization of Personalized Amlodipine Dosing Strategies for Children Based on Pharmacokinetic Data from Chinese Male Adults and PBPK Modeling

**DOI:** 10.3390/children8110950

**Published:** 2021-10-22

**Authors:** Xiaolu Han, Xiaoxuan Hong, Xianfu Li, Yuxi Wang, Zengming Wang, Aiping Zheng

**Affiliations:** 1 State Key Laboratory of Toxicology and Medical Countermeasures, Beijing Institute of Pharmacology and Toxicology, 27th Taiping Road, Haidian District, Beijing 100850, China; hanxiaolu921007@163.com (X.H.); hongxiaoxuan1216@163.com (X.H.); xiaofu0924@163.com (X.L.); 2 Troops 32104 of People’s Liberation Army of China, Alashan League 735400, China; 3 Shanghai PharmoGo Co., Ltd., 3F, Block B, Weitai Building, No. 58, Lane 91, Shanghai 200127, China; easonwang@pharmogo.com

**Keywords:** amlodipine, pediatric preparations, dosage optimization, PBPK model, hypertension, model-informed drug development

## Abstract

For children, a special population who are continuously developing, a reasonable dosing strategy is the key to clinical therapy. Accurate dose predictions can help maximize efficacy and minimize pain in pediatrics. **Methods:** This study collected amlodipine pharmacokinetics (PK) data from 236 Chinese male adults and established a physiological pharmacokinetic (PBPK) model for adults using GastroPlus™. A PBPK model of pediatrics is constructed based on hepatic-to-body size and enzyme metabolism, used similar to the AUC_0-∞_ to deduce the optimal dosage of amlodipine for children aged 1–16 years. A curve of continuous administration for 2-, 6-, 12-, 16-, and 25-year-olds and a personalized administration program for 6-year-olds were developed. **Results:** The results show that children could not establish uniform allometric amplification rules. The optimal doses were 0.10 mg·kg^−1^ for ages 2–6 years and −0.0028 × Age + 0.1148 (mg/kg) for ages 7–16 years, r = 0.9941. The trend for continuous administration was consistent among different groups. In a 6-year-old child, a maintenance dose of 2.30 mg was used to increase the initial dose by 2.00 mg and the treatment dose by 1.00 mg to maintain stable plasma concentrations. **Conclusions:** A PBPK model based on enzyme metabolism can accurately predict the changes in the pharmacokinetic parameters of amlodipine in pediatrics. It can be used to support the optimization of clinical treatment plans in pediatrics.

## 1. Introduction

With the changes in people’s dietary patterns and living habits in recent years, the incidence of essential hypertension in children is increasing year by year [1]. According to the China Cardiovascular Disease Report, 3–4% of Chinese children had hypertension by the end of 2015, with an average annual growth rate of 0.47% [2]. Studies of pediatric drug safety data have shown that calcium channel blockers (CCBs) are the front-line medication for children with hypertension. Currently, amlodipine is the only CCB approved by the FDA, since 2004, for the treatment of hypertension in children [3]. The clinical data on amlodipine in pediatrics are limited and focused on efficacy and safety [4,5,6]. To select the right dosage and application strategy in children, pharmacokinetic (PK) information is necessary [6]. In Europe and America, several studies have shown the pharmacokinetic differences of amlodipine between children and adults [7,8,9], while few studies have reported on its use in Chinese children.

The ages of children have a wide range, and children are continuously growing and developing. Traditional calculation methods have been based on body weight (e.g., Clark’s rule), age (e.g., Young’s rule), and body surface area (e.g., mg/m^2^) [10,11]. These ignore the differences in the organ maturation rates, blood flow, body composition, mechanisms of drug elimination and metastasis, decreased liver and kidney function due to organic lesions, and ethnic and genetic differences in a given population. A series of Model-Informed Drug Development (MIDD) strategies, including Physiology-Based Pharmacokinetic Modeling, can help optimize the dose [12] and predict the PK parameters [13] in pediatrics. With the development of new technology, such as 3D printing technology, mini-tablets, and other multi-dose pharmaceutical means, the problem of personalized drug treatments was solved at the technical level, which opened up a new area of pediatric drug research and development.

In this study, the starting dose of amlodipine in Chinese children was predicted based on existing PK data from healthy Chinese male adults, the allometric scale law, and the developmental level of clearance and the safety of drugs for different age groups of pediatric population is considered. This study is also the first in which PBPK modeling and simulations were used instead of the amlodipine weight dose estimation method and in which a personalized dosing strategy was developed, making this study a future guideline for individualized treatments of hypertension in Chinese children.

## 2. Methods

### 2.1. Physicochemical and PK Properties of Amlodipine

Amlodipine (molecular formula: C_20_H_25_ClN_2_O_5_) [14] is slightly soluble in water, has a plasma protein binding rate at about 98%, and has a high bioavailability (64–90%) [15]. Amlodipine is not affected by fasting, food, and grinding. After oral administration, it is gradually absorbed from the gastrointestinal tract and widely distributed among whole-body tissue [16]. The distribution volume (V_d_) is about 21 L/kg; the maximum plasma concentration is 6–12 h; and the elimination half-life is long, about 30 to 50 h [16]. After 7–8 days of continuous administration, a blood concentration steady state was reached [13]. Amlodipine is inactivated by liver metabolism, and the metabolites have no obvious calcium antagonist effect: 75% of the metabolites are excreted by the kidneys, 5% of the metabolites are excreted in their original form in urine, and 20–25% are excreted in stool. For special patients with renal failure, amlodipine usually does not need to be adjusted or supplemented [11,17,18,19,20]. For patients with liver damage or who are older, the dose needs to be reduced [21].

### 2.2. The Flow of Dosage Predictions for Children Based on the PBPK Model

The GastroPlus™ (Version 9.8, Simulations Plus Inc., Lancaster, CA, USA) software was used to conduct the modeling and simulations. The ACAT™ module predicts the rate and extent of oral absorption in the gastrointestinal tract [22], whereas the PBPK Plus™ module incorporates a whole-body PBPK model that utilizes differential equations to mathematically describe the drug distribution and elimination for all major tissue compartments [23]. Human physiologies were generated using the program of internal Population Estimates for Age-Related (PEAR™) Physiology module [24]. 

**Figure 1:**
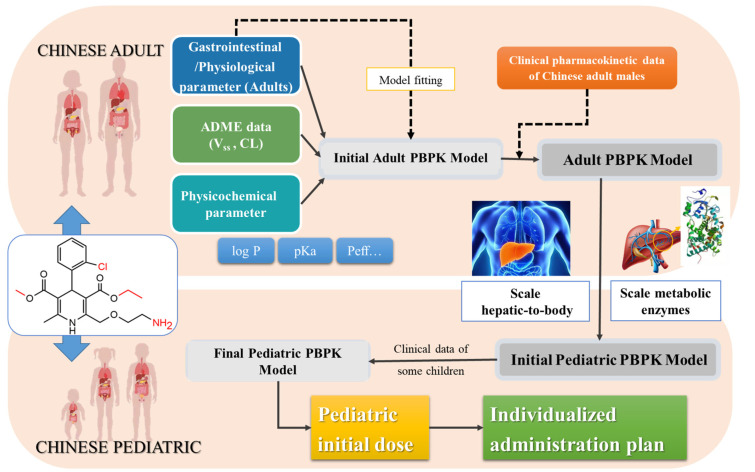
Workflow for the development of the PBPK model to predict dosages.

The workflow involves building and verifying a PBPK model of adults, building and verifying a PBPK model of children, predicting the optimal dosage in children, and formulating individualized administration plans. The details of the strategy are shown in Figure 1. The first step involves building a basic PBPK model for healthy adults compared with observed in vivo data, constantly refining sensitive parameters. Second, the pediatric PBPK was established from the adult PBPK by changing the physiological parameters, predicted using population estimates of the age-related physiology (PEAR™) and ACAT™ modules [25]. The drug-dependent parameters were kept the same as in the adult PBPK. Third, the PBPK models were established and validated in the pediatric population, which accounts for physiological changes that certainly affect the PK of amlodipine. We then further predicted the dosages given to different pediatric populations. Finally, the predicted PBPK model was used to formulate individualized administration plans for target groups.

### 2.3. Amlodipine PBPK Model for Chinese Male Adults

The PBPK model used a database of physiological and physiological parameters from Chinese male adults. The total clearance rates considered in this model stemmed from the sum of those from the kidney and liver, of which the liver clearance rate accounted for 90%. The model calculated the steady-state distribution volume (*V_SS_*) according to Equation (1) [26]. This value is identical to that obtained from the study by J.K. Faulkner et al. on venous PK data (single dose, 10 mg) in adult American males [27].

(1) $$V_{ss}=V_{p}+V_{e}+E:P+\sum V_{t}\times K_{pt}\times \left(1-ER_{t}\right)$$

where $V_{p}$ is the plasma volume, $V_{e}$ is the red blood cell (RBC) volume, E:P is the RBC plasma concentration ratio, $V_{t}$ is the tissue volume, $K_{pt}$ is the tissue plasma partition coefficient, and $ER_{t}$ is the tissue extraction rate.

The model input parameters are shown in Table 1. A time–concentration curve for oral administration of 10 mg within 196 h was obtained using a simulation, and a PK analysis was performed on the simulated profile using software.

By collecting clinical data and building an adult model, the authors of [29] found that individual variables of amlodipine are obvious. The stability of the model can be improved by increasing the amount of clinical data collected from the Chinese population. Based on this, this study modified the clinical pharmacokinetic data [30,31,32,33,34,35,36,37,38,39,40,41] of 236 Chinese male adults administered amlodipine orally on the basis of simulation and corrected the simulation by calculating the mean pharmacokinetic parameters (AUC_0-∞_) of the clinical data. Detailed information on the clinical PK data of Chinese male adults used for the modeling calibration is listed in Table 2. The specific parameters of the modified model are as follows: bioavailability (F) 84.488%, t_max_ 5.7671 h, c_max_ 5.4587 ng/mL, and AUC_0-∞_ 272.70 ng/h·mL. The confidence interval of the simulation is 90%.

The model fitting degree was evaluated by PE, and the calculation is shown in Equation (2). If PE < 10%, the fitting degree is good and the prediction model is reliable and effective [42,43,44].

(2) $$\%\;\mathrm{PE}=\frac{\left|Predicted-Observed\right|}{Observed}\times 100\%.\;$$

### 2.4. Amlodipine PBPK Model for Chinese Pediatrics and Dose Prediction

Weight, age, and organ function are three important covariates that affect PK variation in the pediatric population. In order to identify the key factors that influence the variation in amlodipine pharmacokinetics in children, to evaluate the key factors in children’s metabolism, and to establish a relationship for dose calculation, the initial dose of amlodipine was calculated by means of an adult body weight conversion and the PEAR physiology module building PBPK model. During the modeling process, specific data of children of different ages came from built-in data from the GastroPlus^™^ and derived data, as shown in Table S1.

#### 2.4.1. Dose Prediction by Adult Body Weight

In the instructions of Norvasc^®^, the recommended starting dose for healthy adults is 5.00 mg for adults with a body weight of 70 kg. This dosage was converted to standard doses for children of different body weights. Equation (3) is used to calculate the dose for children based on body weight:

(3) $$\mathrm{Doses}\;\mathrm{in}\;\mathrm{children}\left(\frac{\mathrm{mg}}{\mathrm{kg}}\right)=\frac{\mathrm{Adult}\;\mathrm{dose}\;}{\mathrm{Adult}\;\mathrm{weight}\;}\times \mathrm{weight}\;\mathrm{in}\;\mathrm{children}=0.07\;\mathrm{mg}/\mathrm{kg}$$

where the weight of children in China is the default value of GastroPlus^™^.

#### 2.4.2. Dose Prediction by PBPK Model

As the amlodipine clearance capacity and methods in different populations are very different, accurate calculation of the clearance (CL) has a great impact on the prediction of PK in vivo. Amlodipine is mainly based on liver metabolism. Therefore, the estimation methods of drug metabolism in this model are mainly as follows:

(1) Clearance by hepatic-to-body size

According to the study by Johnson et al. [5], the change in liver volume from a neonate to an adult is in accordance with a certain law. Infants have a larger hepatic-to-body size compared with older children and adults [5]. It is easy to infer that the CL is directly related to hepatic-to-body size. Liver density remains constant at different age stages, 1.07 g/mL according to calculations. The liver volume is based on the data of Chinese children at different ages obtained from GastroPlus^™^. The standard CL was 29.20 L/h for adults aged 25 years, and the CL for children was calculated using a conversion according to their hepatic-to-body size. Then, the CL was added to the population PBPK model of children of different ages to obtain the predicted doses.

(2) Clearance by hepatic microsomal enzyme expression

When building a PBPK model for pediatrics, the rate that the liver metabolizes amlodipine must be considered, while the intestinal metabolism is negligible. In liver metabolism, the contribution of the CYP 3A5 to 3A subfamily is low (about 10%). The unit tissue of 3A4 enzyme expression in children of different ages is the default value used in GastroPlus^™^. The metabolism of amlodipine through 3A4 was only investigated in the PBPK model.

### 2.5. Rationality of Predicting Dose and Personalized Administration Guidance

The 2017 American Academy of Pediatrics “Clinical Practice Guidelines for the Screening and Management of Hypertension in Children and Adolescents” recommends amlodipine doses for outpatient treatment of hypertension in children [45], as shown in Table 3. With reference to this standard, China revised its hypertension prevention and treatment guidelines. An initial dose for children aged 2–6 years of 0.10 mg/kg was used to verify the dose predicted by the PBPK model for pediatrics. Detailed guidance of the amlodipine dose is lacking for children older than 6 years. 

The validated PBPK model was used to predict the simulation of continuous-dose 10-day time curves for different age groups. The simulations are as follows: Simulation 1, a child aged 2 years old (1.34 mg, q.d.); Simulation 2, a child aged 6 years old (2.30 mg, q.d.); Simulation 3, a child aged 12 years old (3.47 mg, q.d.); Simulation 4, a child aged 16 years old (4.15 mg, q.d.); and Simulation 5, an adult aged 25 years old (5.00 mg, q.d.).

Personalized administration plans can help children quickly reach a steady-state plasma concentration in a short time. Based on this, personalized administration plans were incorporated into the simulation. Simulation 6 was based on Simulation 2, but the initial dose was adjusted to achieve a stable plasma concentration quickly. Simulation 7 was based on Simulation 6, but after one missed dose on the fourth day (72 h), the supplement dose on the fourth day (96 h) was predicted to ensure that the blood drug concentration quickly recovered to steady state.

## 3. Results

### 3.1. Amlodipine PBPK Model for Chinese Male Adults

**Figure 2:**
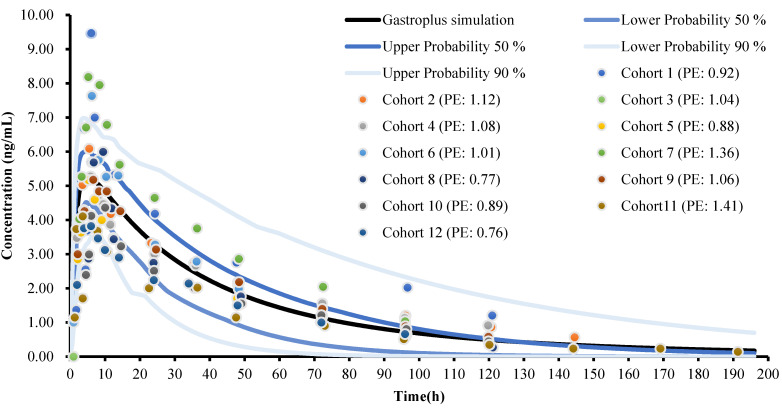
PBPK model of amlodipine administered orally in Chinese male adults and a summary of the clinical data [30,31,32,33,34,35,36,37,38,39,40,41]. (Cohort 3 [32] did not provide detailed drug-time curve data).

The PBPK model was used to optimize the pharmacokinetic curve for 10.00 mg of amlodipine in Chinese male adults, as shown in the solid black line in Figure 1. Using the population simulator output, the drug-time curve distribution of 100 Chinese (male and female) aged 19–40 with a weight of 58–80 kg was simulated, and the simulation time was 196 h. The blue and light blue solid lines in Figure 2 show the simulation at 90% and 50% population simulation probabilities, respectively. The scattered dots with different colors represent the clinical data from the literature and are distributed around the simulation curve. The AUC_0-∞_ (272.70 ng/h·mL) of the optimized PBPK model was close to the median of the clinical data (287.57 ng/h·mL), and the optimized model could be used for a PK simulation of amlodipine.

### 3.2. Prediction of Amlodipine Dose in Chinese Pediatric

**Figure 3:**
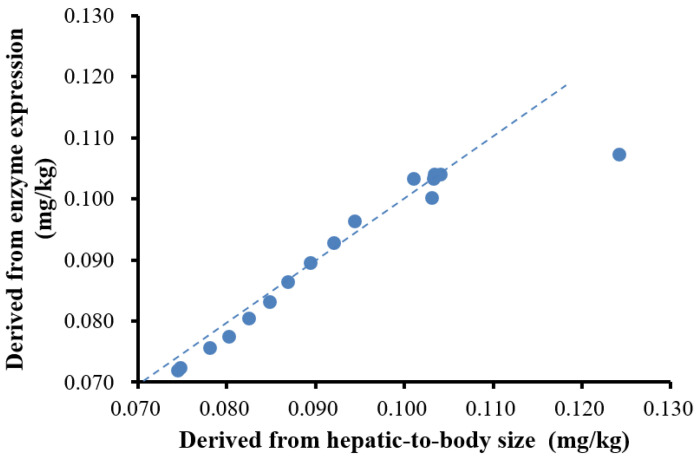
Physiology-based pharmacokinetic (PBPK) predicted dosages derived from the relative liver weight vs. those derived from enzyme expression (1–16 years old).

The doses in children predicted using different methods are shown in Table 4. As can be seen from the results, the doses predicted by constructing the PBPK model based on hepatic-to-body size and enzyme expression levels was higher than the doses derived according to weight (0.70 mg/kg), which was not linear. Moreover, the overall trend of the PBPK model based on hepatic-to-body size and enzyme expression levels is consistent, and the linear fit of the two is shown in Figure 3. The two algorithms of PBPK were evaluated and found to differ in their derived doses only for younger children, especially 1-year-old children. This result is due to the fact that the hepatic drug enzyme expression level of young children is still developing. The metabolic rate of amlodipine was the same in adults and children, but there were differences in the hepatic-to-body size and enzyme expression levels. The liver volume of young children is relatively large, but the enzyme expression is still incomplete [46]. The liver drug enzyme expression tends to be stable after 6 years of age, and the hepatic-to-body size tends to be stable after 14 years of age (this conclusion was based on an analysis of unit tissue enzyme expression in children of different ages built into GastroPlus™). Drug metabolism is the comprehensive effect of body change. Therefore, the construction of a PBPK model requires comprehensive consideration of various factors of drug metabolism and child development.

### 3.3. Amlodipine Extrapolation Rules for Children

**Figure 4:**
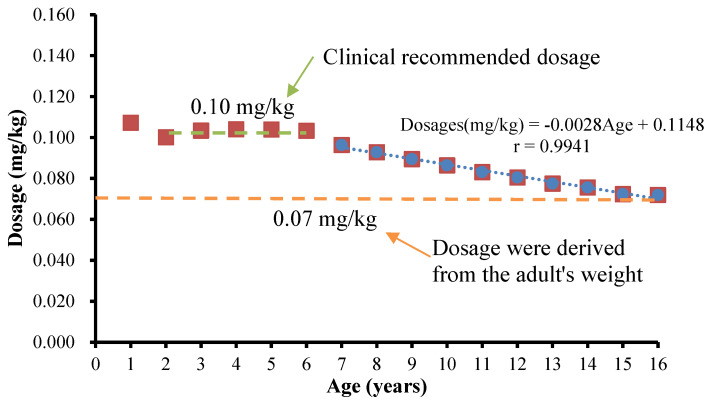
The relationship between the dosage derived from the PBPK model constructed by enzyme expression and the recommended dosage derived by body weight.

Combined with the prediction of amlodipine dose, it was found that a single dose could not meet the extrapolation rules of children. According to the results, a dose derived from PBPK based on enzyme expression was more reliable. The weight-adjusted amlodipine dose per kilogram for younger children aged 2–6 years was significantly higher than that for older children, indicating that a higher dose is required for treatment of young children with amlodipine, which is consistent with clinical data [11,17,18,47,48]. In this study, the high dose range of amlodipine was determined according to the PBPK model. The clinical dose of amlodipine ranged from 0.07 mg/kg to 0.11 mg/kg. A detailed administration strategy is shown in Figure 4. The dose for children aged 2–6 years was similar to the clinical recommendation of 0.10 mg/kg, and the doses were in the range of 1.34–2.30 mg. A linear formula can be established for age and dosages (mg/kg) for children aged 6–16 years: Dosages (mg/kg) = − 0.0028 × Age + 0.1148, r = 0.9941. The doses for children over 16 years of age were 0.07 mg/kg, the same as that for adults.

**Figure 5:**
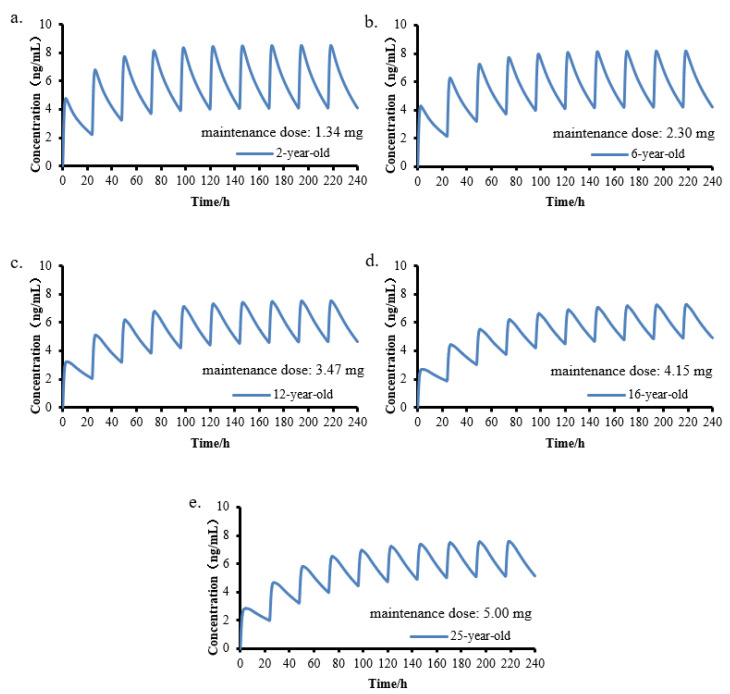
The curve simulation diagram of continuous administration for 240 h in different age groups. (**a**). Simulation 1, a child aged 2 years old, 1.34 mg, q.d.; (**b**). Simulation 2, a child aged 6 years old, 2.30 mg, q.d.; (**c**). Simulation 3, a child aged 12 years old, 3.47 mg, q.d.; (**d**). Simulation 4, a child aged 16 years old, 4.15 mg, q.d.; (**e**). Simulation 5, an adult aged 25 years old, 5.00 mg, q.d.).

The PBPK model was used to predict the plasma concentration given once daily for 10 days in a child aged 2 years old (1.34 mg), a child aged 6 years old (2.30 mg), a child aged 12 years old (3.47 mg), a child aged 16 years old (4.15 mg), and an adult aged 25 years old (5.00 mg). The curves of continuous administration for 240 h are shown in Figure 5. The results showed all simulations in vivo; the elimination of amlodipine can be captured well using the PBPK model. The simulations showed the same pharmacokinetic characteristics: the plasma concentration reached homeostasis after 7–8 days of continuous administration. AUC_0-∞_ had a good predictive value in all simulations (the prediction–measured ratio was 0.96–1.05), but t_max_ appeared earlier in younger children. A similar trend has been observed in patients who are older in some clinical studies, which is suspected to be related to an imperfect absorption barrier.

### 3.4. Personalized Administration Guidance

**Figure 6:**
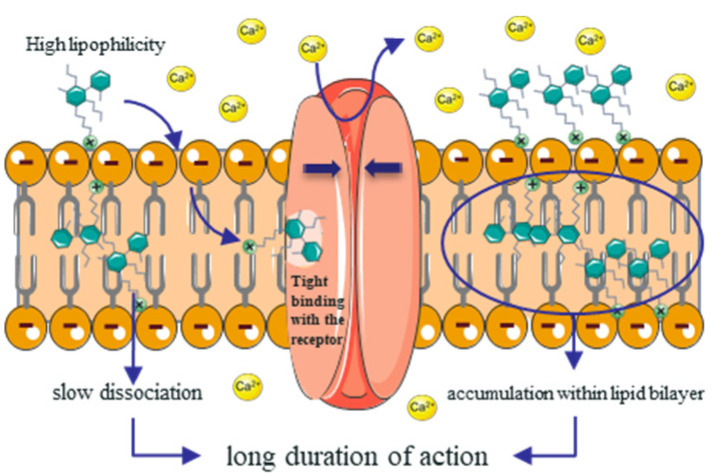
Diagram of the interaction of amlodipine with the dihydropyridine receptors, and the cumulative effect.

Unlike most CCBs, amlodipine has a concentration of more than 90% ionized at the physiological pH, and has a positive charge [5]. The increased ionization of amlodipine increased the concentration of the drug in the lipid bilayers of the cell membrane and produced an accumulative effect, shown in Figure 6. This effect causes amlodipine to have longer t_1/2_ and t_max_, and a stable plasma concentration is required after 7–8 days of continuous administration. Therefore, the development of personalized and flexible drug administration strategies can quickly and stably regulate blood pressure, which is more conducive to protecting important organs such as blood vessels, the heart, the brain, and the kidneys.

**Figure 7:**
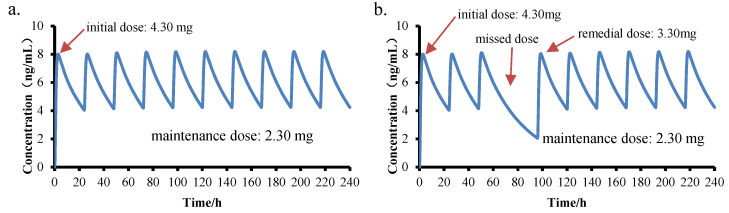
The curve simulation diagram of a personalized administration plan of amlodipine for 6-year-old children. (**a**). Simulated 6: Initial dose: 4.30 mg, maintenance dose: 2.30 mg, q.d.; (**b**). Simulated 7: On the basis of simulation 6, 3.30 mg was taken on the fifth day after missing the fourth day).

The construction of PBPK model has great guiding significance for children’s personalized drug therapy. In PBPK model, the initial dose, maintenance dose, and remedial dose were changed to minimize the fluctuation in blood concentration. According to the simulation results, on the basis of the PBPK model with a dose of 2.30 mg (q.d.) for a 6-year-old Chinese child (weight 22.26 kg), an initial dose of 4.30 mg can quickly reach the steady-state concentration, shown in Figure 7a. Patients with hypertension who frequently miss medication will have a decreased blood pressure compliance rate. In the case of missed administration or late administration, the appropriate remedial dose can be explored using the PBPK model. Assuming that the child missed administration once on the fourth day (time: 96 h), 3.30 mg should be administered on the fifth day (120 h) for remedial use, which can ensure that the plasma concentration reaches a steady state, as shown in Figure 7b.

## 4. Discussion

Currently, only some clinical data of amlodipine in children are available. In the absence of indications or clinical data in children, rational use of adult clinical trial data can avoid unnecessary clinical trials in children. PBPK models have been proven to be important tools in drug development and regulatory evaluation [49] and have been used more and more in the field of pediatrics to predict dosages [49,50]. Age-related physiological PBPK model construction can quantify the relationship with the continuous growth of children, especially in describing the in vivo pharmacokinetic characteristics of unspecified populations, providing bioavailability/bioequivalence data and providing references and a basis for bridging clinical treatment characteristics.

In this study, a comprehensive analysis of all of the available information and data on amlodipine, including differences in organ function and pharmacological characteristics among people of different ages, non-clinical experimental data, clinical effectiveness and safety differences, etc., found that amlodipine’s antihypertensive effect was similar in the disease course, treatment response, and in vivo exposure–response relationship of the drug in adults and children. Second, an adult PBPK model was successfully established using the collected clinical data. Then, a pediatric PBPK was established from the adult PBPK by changing the physiological parameters. The extrapolation rules of amlodipine from adults to children were established, and a time curve of continuous administration of amlodipine in a specific age group was simulated. Finally, in the case of 6-year-olds, the PBPK model demonstrates how to provide personalized drug regimens for specific populations.

In the adult model, it was found that amlodipine was affected by the variation in physiological parameters such as enzyme expression level and body weight among different individuals, and PK changed significantly. In order to reduce the variation and to improve the stability, the current model was adjusted based on the average exposure of 236 subjects. From the results, the PK of amlodipine in vivo was significantly affected by weight, hepatic-to-body size, and enzyme expression. Studies on the pharmacokinetics of amlodipine in children have shown that there may be significant pharmacokinetic differences between adults and younger pediatric populations [7,12,51].

The predicted dose using the PBPK model of this study appears to support these clinical observations. According to the results of the dose prediction, the amlodipine pharmacokinetic parameters were similar to those in adults for children with body weights comparable to adults. However, for children with low body weights, namely toddlers and young children younger than 6 years of age, the amlodipine pharmacokinetic parameters were significantly different, requiring greater weight-adjusted doses than in the older subjects. It is speculated that the proportion of liver-to-body size in young children is higher than that in adults, that the expression of liver drug enzymes gradually matures and tends to be stable after 6 years of age, and that the growth rate of the liver is lower than that of the body. With an increase in age, the number of liver cells and the ability to metabolize related drugs are indirectly affected.

According to the results of the study, a steady-state plasma concentration of amlodipine in the treatment of hypertension can be achieved after 7–8 days of medication. In addition, long-term use of amlodipine may cause occasional missed doses. In clinical practice, the PBPK model can be adjusted according to the child’s own conditions (such as age, weight, gender, race, genotype detection, liver and kidney diseases, etc.) to develop personalized drug administration strategies to guide clinical medication. This strategy can minimize the fluctuation in PK parameters caused by dose prediction errors, can effectively control blood pressure, and can protect organ functions. Although the study could not avoid differences in race, gender, and genetic diversity, it still provides theoretical guidance and a basis for clinicians to make effective initial doses, maintenance doses, and remedial doses after a missed dose in full and careful consideration. The significance of this study lies not only in the formulation of an effective extrapolation rule, but also in the formulation of personalized amlodipine protocols for clinical practice.

## 5. Conclusions

In this paper, an adult PBPK model for Chinese male adults was constructed using GastroPlus^TM^, and a children PBPK model was extrapolated according to the growth and organ maturity of children. The doses of amlodipine in children were predicted based on the constructed children model, and an extrapolation rule suitable for Chinese children was constructed. In the follow-up studies, continuous-dose curves of 2-, 6-, 12-, 16-, and 25-year-old patients were simulated; personalized strategies for rapidly reaching blood concentrations and remedial for missed doses were used; and two personalized drug delivery strategies were developed for 6-year-old patients. As amlodipine has a long-term sustained-release characteristic due to its accumulation effect, stable plasma concentrations are achieved 7–8 days after continuous administration. Most instances of hypertension in young children were secondary, long-term medication is needed, and rapid and stable adjustment of blood pressure is more conducive to the protection of organs. On the basis of the safe dose predicted by the PBPK model established in this study, a reasonable increase in the first dose can avoid a long cycle of adjustment to amlodipine; can protect systemic blood vessels, the heart, the brain, the kidneys, and other important organs; can improve drug compliance in children; and can provide a reference for the individualized treatment of hypertension in children.

## Figures and Tables

**Table 1:** Physicochemical and biopharmaceutical properties of amlodipine used in the PBPK models.

Parameter	Data
Molecular weight (g/mole)	408.88 ^a^
pKa weak base	9.1 ^a^
Partition coefficient	1.34 (pH = 7.8) ^b^
Solubility (mg/mL)	4.23 (pH = 4.0) ^b^
Mean particle radius (μm)	4.47 ^b^
log P (n-octanol: water)	2.96 ^a^
Peff (human jejunal permeability)	0.45 × 10^−4^ (Caco-2) ^c^
Mean precipitation time (sec)	900 ^d^
Drug particle density (g/mL)	1.2 ^d^
Blood plasma concentration ratio (%)	6.5 ^c^
Unbound percent in plasma (%)	2.0 ^e^

^a^. A guideline for adjustments of the dosage of amlodipine after co-administration [28]; ^b^. An experimental value; ^c^. The fitting values, in which Peff is based on the t_max_ oral absorption percentage, the blood plasma concentration ratio was optimized according to V_ss_, and the steady distribution volume of the veins was determined; ^d^. GastroPlus™ default values; ^e^. Clinical pharmacokinetics of amlodipine [15].

**Table 2:** Clinical pharmacokinetic data of amlodipine in Chinese male adults.

Cohort/Ref.	Dosage *	Gender	Mean Age	Number	Weight (kg)	t_max_ (h)	c_max_ (ng/mL)	t_1/2_ (h)	AUC_0-__∞_ (ng/h·mL)
1 [30]	Oral 10 mg	Male	24.10	16	64.00 ± 6.00	7.06 ± 0.73	7.30 ± 2.91	35.81 ± 5.14	249.89 ± 59.24
2 [31]	Oral 10 mg	Male	22.52	20	70.17 ± 6.10	5.95 ± 1.23	6.13 ± 1.35	40.02 ± 7.49	306.4 ± 91.3
3 [32]	Oral 10 mg	Male	-	20	67.00 *	6.90 ± 1.70	8.00 ± 2.00	26.50 ± 6.20	283.1 ± 56.2
4 [33]	Oral 10 mg	Male	35.60	22	64.00 ± 5.50	7.64 ± 4.35	5.34 ± 3.09	47.87 ± 12.67	294.8 ± 216.9
5 [34]	Oral 10 mg	Male	22.00	18	71.3 ± 2.49	5.83 ± 1.54	5.55 ± 1.97	30.88 ± 4.51	240.3 ± 82.3
6 [35]	Oral 10 mg	Male	21.50	18	60~80	6.00 ± 0.00	7.60 ± 2.50	32.50 ± 7.10	274.6 ± 93.8
7 [36]	Oral 10 mg	Male	20.50	20	65.55 ± 6.87	6.40 ± 1.00	9.57 ± 0.96	31.77 ± 5.07	371.6 ± 45.8
8 [37]	Oral 10 mg	Male	23.80	18	63.90 ± 5.50	7.00 ± 1.00	6.27 ± 2.41	32.60 ± 4.50	209.4 ± 55.3
9 [38]	Oral 10 mg	Male	23.90	18	61.20 ± 1.30	7.90 ± 2.94	6.02 ± 1.45	42.00 ± 13.30	287.9 ± 84.0
10 [39]	Oral 10 mg	Male	25.00	18	67.78 **	8.20 ± 2.20	5.00 ± 2.20	49.00 ± 24.00	243.0 ± 89.0
11 [40] ***	Oral 5 mg	Male	-	24	67.67 **	6.38 ± 2.33	4.37 ± 1.14	43.22 ± 16.63	192.8 ± 62.7
12 [41] ***	Oral 5 mg	Male	21.00	24	60.80 ± 7.60	7.30 ± 6.30	2.23 ± 0.71	39.57 ± 15.40	103.2 ± 39.0
Summary **** PE = 4.69%					66.59	6.86	6.64	39.15	287.57

* The data were collected using Pfizer’s Norvasc^®^ 5 mg as a reference preparation. ** GastroPlus™ default values; *** Amlodipine fits the linear pharmacokinetics profile after multiplying C_max_ and the AUC by two when modeling; **** The data are modified by reserving two decimal places, and the summarized results are summarized as the original data.

**Table 3:** Dosage recommendations of amlodipine for antihypertensive drug treatments in outpatient hypertensive patients [45].

Age	Initial Dosage	Maximum Dosage	Dosing Intervals
1–2	0.10 mg/kg	0.6 mg/kg (Maximum 5.00 mg/d)	q.d.
3–6			
>6	2.50 mg	10.00 mg	q.d.

**Table 4:** Dosage derivation of amlodipine administered orally in a Chinese pediatric population.

Age	Prediction by Body Weight *	Prediction Dose by PBPK Model			
		Hepatic-to-Body Size		Enzyme Expression	
	mg	mg	mg/kg	mg	mg/kg
1	0.71	1.24	0.12	1.07	0.11
2	0.96	1.38	0.10	1.34	0.10
3	1.13	1.64	0.10	1.64	0.10
4	1.27	1.85	0.10	1.87	0.11
5	1.40	2.03	0.10	2.08	0.11
6	1.59	2.25	0.10	2.30	0.10
7	1.85	2.45	0.09	2.50	0.10
8	2.08	2.68	0.09	2.70	0.09
9	2.31	2.90	0.09	2.90	0.09
10	2.56	3.12	0.09	3.10	0.09
11	2.82	3.35	0.08	3.28	0.08
12	3.08	3.56	0.08	3.47	0.08
13	3.34	3.76	0.08	3.63	0.08
14	3.61	3.95	0.08	3.82	0.08
15	3.87	4.05	0.07	3.92	0.07
16	4.12	4.30	0.07	4.15	0.07

* The weight of the pediatric population of different ages was the default value from GastroPlus™, Conversion is based on a 0.07 mg/kg dose.

## Supplementary Materials

The following are available online at https://www.mdpi.com/article/10.3390/children8110950/s1, Table S1: Data from the Chinese pediatric population were used in the model
