# Supplementary material for: Optimization of Personalized Amlodipine Dosing Strategies for Children Based on Pharmacokinetic Data from Chinese Male Adults and PBPK Modeling
Source: Children (Basel). 2021 Oct 22;8(11):950. doi: 10.3390/children8110950 (PMC8618961; doi:10.3390/children8110950)
Supplement: Supplementary file 1 [file children-08-00950-s001.zip › children-1405136-supplementary.pdf]

During the modeling process, specific data for children of different ages were further supplemented, including built-in data from the GastroPlus™ and derived data.

Table S1. Data from the Chinese pediatric population were used in the model.

Age	Weight (kg)*	height	Hepatic volume (ml)	Hepatic density (g/ml)	Hepatic weightg (g)	Hepatic-to-body (%) *	Clearance rate (l/hr)	Enzyme expression (mg·enz/g·tissue) *
1	9.98	74.15	319.28	1.07	341.63	3.42%	7.44	0.175
2	13.38	85.74	367.31	1.07	393.02	2.94%	8.55	0.200
3	15.87	94.99	432.75	1.07	463.04	2.92%	10.08	0.209
4	17.78	103.08	487.11	1.07	521.21	2.93%	11.35	0.214
5	19.63	110.49	537.31	1.07	574.92	2.93%	12.51	0.217
6	22.26	117.69	592.87	1.07	634.37	2.85%	13.81	0.219
7	25.95	122.47	644.19	1.07	689.28	2.66%	15.00	0.220
8	29.1	129.31	702.13	1.07	751.28	2.58%	16.35	0.220
9	32.42	135.9	760.14	1.07	813.35	2.51%	17.70	0.220
10	35.88	142.18	817.63	1.07	874.87	2.44%	19.04	0.220
11	39.47	148.09	874.22	1.07	935.42	2.37%	20.36	0.220
12	43.13	153.57	929.07	1.07	994.10	2.30%	21.64	0.220
13	46.84	158.55	981.64	1.07	1050.35	2.24%	22.86	0.220
14	50.54	162.97	1031.10	1.07	1103.28	2.18%	24.02	0.220
15	54.18	166.78	1049.82	1.07	1123.31	2.07%	24.45	0.220
16	57.71	169.91	1116.00	1.07	1194.12	2.07%	25.99	0.220

* Default value of GastroPlus™.
